# Prevalence of Organ-Specific Autoimmunity in Patients With Type 1 Diabetes Mellitus

**DOI:** 10.7759/cureus.38855

**Published:** 2023-05-10

**Authors:** Ahmad Alam, Surya K Singh, Ritesh Kumar

**Affiliations:** 1 Endocrinology, Diabetes and Metabolism, Rajiv Gandhi Centre for Diabetes and Endocrinology, Jawaharlal Nehru Medical College Hospital (JNMCH) Aligarh Muslim University, Aligarh, IND; 2 Endocrinology, Diabetes and Metabolism, Department of Endocrinology and Metabolism, Institute of Medical Sciences (IMS) Banaras Hindu University, Varanasi, IND

**Keywords:** organ-specific autoimmunity, ttgab, pca, gada, tpoab, type 1b diabetes, type 1 diabetes

## Abstract

Introduction: Type 1 diabetes mellitus (T1DM) is associated with other autoimmune disorders that are characterized by presence of organ-specific autoantibodies. The present study was undertaken to assess the prevalence of organ-specific autoantibodies among newly diagnosed T1DM subjects of India and to study its relationship with glutamic acid decarboxylase antibody (GADA). We also compared the clinical and biochemical parameters in GADA-positive and -negative T1DM subjects.

Methods: In a hospital-based cross-sectional study, we studied 61 patients with newly diagnosed T1DM ≤ 30 years of age. T1DM was diagnosed on the basis of acute onset of osmotic symptoms with or without ketoacidosis, severe hyperglycaemia [blood glucose > 13.9 mmol/l (>250 mg/dl)] and insulin requirement from the onset of diabetes. Subjects were screened for autoimmune thyroid disease (thyroid peroxidase antibody [TPOAb]), celiac disease (tissue transglutaminase antibody [tTGAb]), and gastric autoimmunity (parietal cell antibody [PCA]).

Results: Of the 61 subjects, more than one-third (38%) had at least one positive organ-specific autoantibody. In particular, 13 (21.3%) were found to be positive for TPOAb, nine (14.8%) were positive for tTGAb and 11 (18%) were positive for PCA. GADA was positive in 15 (25%) subjects. The frequency of TPOAb tended to be higher in patients who had GADA positivity compared with those with no circulating GADA (40% *vs.* 15.2%; *p*=0.07). Subjects positive for GADA were also more likely to be PCA positive compared with those who were GADA negative (40 *vs*.10.9%, *p*=0.02). There were no differences in frequency of diabetic ketoacidosis, body mass index, hemoglobin A1C (HbA1c), insulin requirement or fasting C-peptide in GADA-positive and -negative patients.

Conclusion: We support the recommendation for regular screening of organ-specific autoantibodies, in particular TPOAb, tTGAb and PCA in all patients with T1DM. Detection of these autoantibodies at onset may prevent complications associated with delayed diagnosis of these disorders. We also conclude that there is higher frequency of TPOAb and PCA in GADA-positive T1DM patients as compared to negative ones. However, patients with positive GADA had similar clinical and biochemical parameters compared to GADA-negative subjects. Lastly, low GADA positivity in our study cohort as compared to Western populations suggests the heterogenous nature of T1DM in the Indian population.

## Introduction

Type 1 diabetes mellitus (T1DM) is a human leukocyte antigen (HLA)-linked disease caused by an autoimmune destruction of pancreatic islet β-cells that results in progressive insulin deficiency and hyperglycemia. The 1997 American Diabetes Association Expert Committee divided T1DM into type 1A (immune-mediated) and type 1B (idiopathic) [1].

Type 1A diabetes results from cellular-mediated autoimmune destruction of β-cells in the pancreas; abnormal activation of T-cells leads to insulitis and production of antibodies against β-cells, which may constitute useful markers of immune destruction. The most representative autoantibodies detectable in patients with type 1A diabetes are directed against islet cells antigens such as insulin (IAA), insulinoma antigen 2 (IA-2), 65kDa isoform of glutamic acid decarboxylase (GAD-65) and zinc transporter 8 (ZnT8) [2]. Type 1B diabetes is a heterogeneous entity with no islet cell antibodies, in which the reason for β-cell destruction is unknown. A fulminant form of type 1B diabetes with associated exocrine pancreatic involvement, possibly secondary to viral infection, has been described in Japanese [3].

The prevalence of islet cell antibodies varies in relation to patient’s age, disease duration and ethnic origin. In Caucasians one or more islet-antibodies are present in >90% of the patients at the onset of T1DM while idiopathic diabetes is uncommon [4]. On the contrary, low prevalence of autoantibodies has been reported in Asian countries. Prevalence of 14 to 53% glutamic acid decarboxylase antibody (GADA) positivity in T1DM patients has been reported from different studies across India [5-10]. A high frequency of type 1B diabetes (30%) has been reported in Indian children using antibodies against all four major islet-antigens [10].

T1DM is frequently associated with other autoimmune disorders, such as autoimmune thyroid disease (AIT), celiac disease (CD), pernicious anemia, and Addison’s disease. Presence of glutamic acid decarboxylase antibody (GADA) positivity in children and adolescents with T1DM has been strongly related to the development of organ-specific autoimmunity. But the data on prevalence of organ-specific autoantibodies in GADA-negative T1DM are lacking. Type 1B diabetes patients do not have pancreatic autoantibodies but if they have other organ-specific autoantibodies is not known.

Thus, the aim of our study was to evaluate the prevalence of associated organ-specific autoantibodies in subjects with T1DM and to study their relationship with GADA. We also compared the clinical presentation, glycemic status, insulin requirement and C-peptide values in GADA-positive and -negative T1DM patients.

## Materials and methods

Ethics approval

The study was approved by the institutional ethics committee of the Institute of Medical Sciences, Banaras Hindu University (approval number:2020/EC/2150). Informed consent was obtained from parents (age less than equal to 18 years) and patients (age more than 18 years).

Study design

We conducted a hospital-based cross-sectional study of newly diagnosed T1DM patients three to 30 years of age attending the endocrinology clinic and emergency of the University Hospital, Institute of Medical Sciences, Banaras Hindu University between January 2020 and March 2022. T1DM was diagnosed on the basis of acute onset of osmotic symptoms with or without ketoacidosis, severe hyperglycaemia [blood glucose > 13.9 mmol/l (>250 mg/dl)] and insulin requirement from the onset of diabetes.

Data collection

All patients with T1DM were evaluated for age, sex, height, weight, and body mass index (BMI). Weight was measured (in kg) on a beam balance with minimal clothing to the nearest 0.1 kg. Height was recorded (in cm) using a stadiometer with head held in Frankfurt plane to the nearest of 0.1 cm. BMI was computed using the formula weight in kilograms (kg) divided by height in meters squared (kg/m^2^). Age and gender-matched Z scores for height, weight and BMI were calculated using WHO growth charts (less than five years) [11] and Indian Academy of Pediatrics (IAP) growth charts (five to 18 years) [12]. Diabetic ketoacidosis (DKA) was diagnosed based on the following criteria: hyperglycemia over 200 mg/dL, venous pH less than 7.3 or plasma HCO3 less than 15 mEq/L and the presence of ketonuria [13].

Antibody Assays

The antibodies were measured within three months after the initial diagnosis of diabetes. On the day of sample collection, fasting venous blood was drawn and serum was separated and stored in aliquots at -20°C until analyzed.

GADA was measured by the enzyme-linked immunoassay (ELISA) method using a commercial kit (Isletest-GAD; Biomerica Inc., Newport Beach, CA, USA). The intra-assay and inter-assay coefficient of variations (CVs) were <5.4% and <4.6%, respectively. The specificity and sensitivity of the kit were 87.1% and 85.0% respectively. A GADA value of > 1.05 U/mL was considered as positive. Thyroid peroxidase antibody (TPOAb) was measured using a chemiluminescence immunoassay (CLIA) using a Beckman Coulter-DXI 800 (Brea, CA, USA). This assay exhibited a total imprecision of <12% at concentrations ≥0.6 IU/ml. TPOAb values >9 IU/ml were considered as positive. IgA anti-tissue transglutaminase antibody (IgA-tTGAb) was measured by the ELISA method using a commercial kit (Diametra Diagnostics, Spello, Italy). The intra-assay and inter-assay CVs were <6% and <11%, respectively. An IgA-tTGAb value of >20 AU/ml was considered as positive. All patients with positive serologic results were offered endoscopic intestinal biopsies to confirm CD. The determination of parietal cell antibodies (PCA) was measured by ELISA method using a commercial kit (Orgentec Diagnostika, Mainz, Germany). The intra-assay and inter-assay CVs were <3.5% and <4.2% respectively. A PCA value of >10 U/ml was taken as positive.

Other Assays

Thyroid-stimulating hormone (TSH), total T4, and total T3 were measured using CLIA using a Beckman Coulter-DXI800. C-peptide determination based on sandwich principle was done by electrochemiluminescence immunoassay (ECLIA) using Elecsys (Roche Diagnostics, Mannheim, Germany) with a lower limit of detection of C-peptide of 0.01 ng/ml. HbA1c was measured by high-performance liquid chromatography (HPLC), ion exchange principle. Blood glucose was measured enzymatically using glucose oxidase peroxidase (GOD-POD) method. The investigations were done on fully automated Mindray SAL 6000 (Shenzhen, China).

Statistical analysis

Quantitative variables were expressed as mean ± standard deviation. They were analyzed using independent sample t-test and Kruskal-Wallis one-way analysis of variance (ANOVA) test. Qualitative variables were expressed as percentage and were analyzed using Pearson chi-square and Fischer Exact test. All the data were analyzed using Statistical Package for Social Sciences (SPSS) version 25.0 (IBM Corp., Armonk, NY, USA). A p-value <0.05 was considered significant

## Results

In the entire study cohort of 61 T1DM, 36 were male. The mean age (± SD) at onset of diabetes was 10.1 ± 4.8 years. Majority of subjects (75%) diagnosed were five to 14 years of age. More than two-thirds of our patients had DKA at presentation. Among all patients with duration of diagnosis less than three months, the prevalence of GADA was 25% (Table 1).

**Table 1:** Baseline characteristics SDS-standard deviation score, BMI-body mass index, DKA-diabetic ketoacidosis, GADA-glutamic acid decarboxylase antibody ^1^ In bracket expressed as percentage

Characteristic	
Number (n)	61
Age at onset (years)	10.1±4.8
Male: Female	1.4:1
Height SDS	-0.4±0.6
Weight SDS	-1.0±0.6
BMI (kg/m^2^)	14.3±1.7
BMI-SDS	-1.1±0.7
DKA at presentation	44 (72.1)^1^
HbA1c (%)	13.3±2.1
Fasting C-peptide (ng/mL)	0.47±0.25
Insulin dose (units/kg)	0.9±0.3
GADA	15 (24.6)^1^

**Figure 1:**
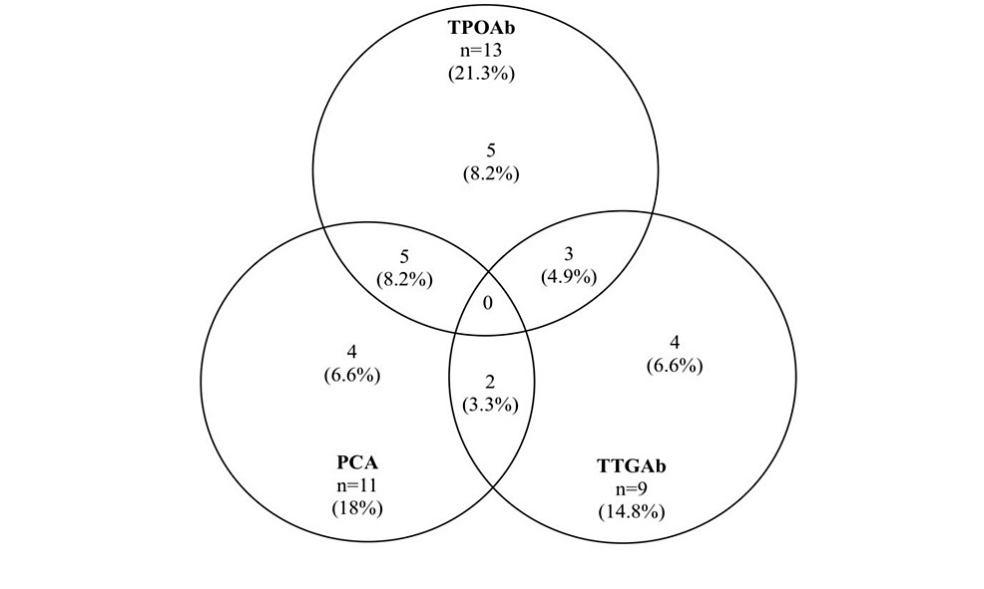
Venn diagram showing frequency of nonislet, organ-specific autoantibodies. Overlapping areas represent individuals positive for more than one autoantibody TPOAb – thyroid peroxidase antibody; PCA – parietal cell antibody; TTGAb – tissue transglutaminase antibody

More than one-third (38%) had at least one positive nonislet organ-specific autoantibody (TPOAb, tTGAb, PCA). In particular, 13 (21.3%) were found to be positive for TPOAb, of whom five (38.4%) had AIT. Nine (14.8%) were positive for tTGAb of whom two had CD confirmed by duodenal biopsy. Eleven (18%) were positive for PCA. Ten subjects (16.4%) were positive for two of the autoantibodies whereas none of them were positive for all three autoantibodies (Figure 1).

GADA-positive patients were older at the age of onset (12.5±4.7 vs 9.4±4.6, p=0.02). Also, subjects positive for GADA were more likely to be PCA positive compared with those who were GADA negative (40 vs.10.9%, p=0.02) (Table 2).

**Table 2:** Comparison between anti-GAD positive and negative patients GADA – glutamic acid decarboxylase antibody, DKA – diabetic ketoacidosis, BMI – body mass index, SDS – standard deviation score, TPO – thyroid peroxidase, tTG – tissue transglutaminase, PCA – parietal cell antibody

	GADA+ (n=15)	GADA- (n=46)	Ch^2^-value	p-Value
Age of onset (years)	12.5±4.7	9.4±4.6		0.02^*^
Male/Female	8 /7	28 /18	0.27	0.76
DKA at presentation	10 (66.7)	34 (73.9)	0.29	0.74
Height SDS	-0.5±0.7	-0.3±0.6		0.32
Weight SDS	-1.1±0.5	-0.9±0.7		0.41
BMI (kg/m^2^)	14.9±1.7	14.2±1.7		0.18
BMI-SDS	-1.1±0.6	-1.1±0.8		0.95
HbA1c (%)	13.0±1.6	13.4±2.3		0.45
C-peptide (ng/ml)	0.45±0.25	0.48±0.26		0.76
Insulin dose (units/kg)	0.9±0.2	0.9±0.3		0.86
TPOAb	6 (40)	7 (15.2)	4.14	0.07
tTGAb	2 (13.3)	7 (15.2)	0.03	1.0
PCA	6 (40)	5 (10.9)	6.49	0.02*

The mean age at diagnosis was 10.1±4.8 in boys and 10.0±4.9 in girls (p=0.93). There were no significant differences in the clinical or biochemical characteristics among boys and girls (Table 3).

**Table 3:** Comparison of clinical and biochemical characteristics in boys and girls with T1DM DKA – diabetic ketoacidosis, BMI – body mass index, SDS – standard deviation score, GADA – glutamic acid decarboxylase antibody, TPO – thyroid peroxidase, tTG – tissue transglutaminase, PCA – parietal cell antibody

	Boys (n=36)	Girls (n=25)	Ch^2^ value	p-value
Age (years)	10.1±4.8	10.0±4.9		0.93
DKA at presentation	26(72.2)	18(72)	0	1
Height SDS	-0.4±0.6	-0.3±0.6		0.42
Weight SDS	-0.9±0.7	-1.0±0.6		0.40
BMI (kg/m^2^)	14.6±1.8	14.1±1.5		0.24
BMI-SDS	-1.0±0.8	-1.3±0.7		0.20
HbA1c (%)	13.3±2.2	13.3±2.1		0.96
Insulin dose (IU/kg)	1.0±0.3	0.9±0.2		0.41
C-peptide (ng/ml)	0.49±0.25	0.43±0.25		0.36
GADA	8 (22.2)	7 (28)	0.27	0.76
TPOAb	7 (19.4)	6 (24)	0.18	0.76
tTGAb	5 (13.9)	4 (16)	0.05	1
PCA	5 (13.9)	6 (24)	1.02	0.5

## Discussion

The mean age at onset of diabetes was 10.1±4.8 years. Majority of our patients diagnosed (75%) were five to 14 years of age. SEARCH International data for T1DM also showed that approximately two-thirds of patients are diagnosed in the five to 14 years age group [14]. The DIAMOND project group reported a steady increase in incidence rate with age up to around 10 to 14 years in 114 populations across the world [15].

The male-to-female ratio in our study cohort was 1.4. Although most autoimmune diseases are more common in females, there appears to be no gender difference in the overall incidence of childhood T1DM. Male to female ratio among children in most populations is 0.9-1 [15]. Dayal et al. in their study (n=647) conducted at Postgraduate Institute of Medical Education and Research (PGIMER), Chandigarh, found a similar male-to-female ratio as seen in our study [16].

The frequency of DKA at diagnosis ranges from 12.8% to 80%, and is lowest in Sweden, the Slovak Republic and Canada and highest in the United Arab Emirates, Saudi Arabia and Romania [17]. In our study, 72% of the patients presented with DKA. In our country, low socioeconomic status and parental ignorance, and limited access to primary and specialized healthcare have been cited as risk factors for DKA at diagnosis [18]. Better disease recognition through improved awareness of diabetes is also supported by the findings that children from families with higher parental education are less likely to present in DKA and having a first-degree relative with diabetes is associated with an up to six-fold decreased risk of DKA at diagnosis [19]. We found no significant difference in frequency of DKA at presentation across different age groups. Similarly, Roche et al. in their study of 283 patients found no significant association between age and presentation in DKA in those aged over two years [20]. However, Dayal et al. found that frequency of DKA decreased with increasing age and younger patients (<five years) were two to three times more likely to present in DKA than older (>15 years) patients [16].

In T1DM patients, other autoimmune diseases are often silent and asymptomatic, and are detected by serological screening. The recent extensive antibody screening has raised awareness of association of T1DM with organ-specific autoimmunity. AIT is the most frequent autoimmune disorder associated with T1DM. Previous studies have reported that 8-54% of T1DM subjects have AIT [21-23], 7-34% have celiac autoimmunity [24-27], 5-34% have parietal cell antibodies [28,29] and 0-3% have adrenocortical antibodies [30,31]. In our study, in agreement with previous studies we have found high prevalence of organ-specific autoimmunity in patients with T1DM. Upon diagnosis 37.7% of patients with T1DM were positive for at least one autoantibody (TPOAb, and/or tTGAb, and/or PCA). Similarly, Triolo et al. in their study of 491 children found that one-third of their patients with recent-onset T1DM were positive for at least one autoantibody [30]. There was no significant difference in the prevalence of TPOAb in boys and girls (19.4% vs. 24%). This is in contradiction to most other studies that have reported a higher prevalence of thyroid antibodies in girls [22,32]. However, Menon et al. in their study of 35 children with T1DM did not find such relation between gender and TPOAb prevalence [23]. The other studies that reported similar prevalence rates in boys and girls were those by Glastras et al. and Hansen et al. [33,34]. In Indian patients with T1DM, a variable frequency of seropositivity of tTG antibody has been reported. Whereas studies from northern and western parts of India have reported a higher prevalence of IgA-tTG positivity ranging from 11-34% [25,27,35], studies from South India have reported a lower prevalence of 5-8% [36]. The reason for this is not clear, but it may be due to the differences in frequency of genetic susceptibility loci or environmental factors such as the age of initiating gluten-containing foods in infancy. There was no significant difference in the prevalence of tTGAb in boys and girls (13.9 % vs. 16%). Barker et al. also found no association between tTGAb positivity and gender [22]. However, Cerutti et al. showed association between female sex and tTGAb positivity in a large cohort of Italian children with T1DM [37]. Data on prevalence of PCA in T1DM in Indian population is sparse. Srikanta et al. found a prevalence of 13% in their study cohort of 110 children with T1DM in North India [38]. Ramachandran et al. found a similar prevalence rate (12.5%) in Southern Indian population [39]. Odugbesan et al. studied the prevalence of auto-antibodies in Indian-Asians residing in the UK and found the prevalence of PCA to be 8% [40]. There was no significant difference in the prevalence of PCA in boys and girls (13.9 % vs. 24%). Riley et al. found a predominance of 63% females vs 37% males in their PCA-positive patients [41]. Other authors, however, did not find such a relation between gender and PCA prevalence [39,42,43].

We also compared the prevalence of organ-specific autoantibody in GADA-positive and -negative patients. The frequency of TPOAb tended to be higher in patients with T1DM who had GADA positivity compared with those with no circulating GADA (40% vs. 15.2%; p=0.07). Barker et al. also observed that subjects positive for GADA were more likely to be thyroid autoantibody positive compared with those who were GADA negative (35% vs. 24%; p=0.008) [22]. Subjects positive for GADA were also more likely to be PCA positive compared with those who were GADA negative (40 vs.10.9%, p=0.02). Also, PCA positivity was associated with TPO positivity (p=0.03). Block et al. also observed that patients with GADA had a higher prevalence of PCA than subjects without GADA (p=0.005) [44]. These observations might be explained by the fact that GAD-65 is not exclusively present in the pancreas but can also be found in brain, ovary, testis, thymus, and in the thyroid and stomach [44,45].

GADA was present in 15 out of 61 (24.6%) subjects in our study. Prevalence of 14 to 53% GADA positivity in T1DM patients has been reported from different studies across India [5-10,31]. Sanyal et al. in their retrospective study of newly diagnosed T1DM children from East India (n=694) found prevalence of GADA to be 24.7% [9]. A high frequency of idiopathic diabetes (30%) has been reported in Indian children using antibodies against all four major islet-antigens [10]. Though we didn’t measure antibodies against other islet-antigens, low GADA positivity in our study corroborates the finding that idiopathic diabetes is substantially more frequent in the Indian population as compared to Caucasians. Low GADA positivity in our study could be due to the immunomodulatory effect of the Bacillus Calmette-Guerin (BCG) vaccine on islet cells [46]. Majority of our patients had BCG vaccination (>90%). Sanjeevi et al. showed that BCG vaccination was associated with decreased GADA positivity (54% in BCG vaccinated vs 100% in BCG non-vaccinated group, p=<0.001) [47]. However, Rohilla et al. observed that successful BCG vaccination did not correlate with islet autoantibody positivity [48].

In our study, GADA-positive patients were older at the age of onset as compared to GADA-negative patients (12.5±4.7 vs. 9.4±4.6, p=0.02). There was no difference in prevalence of GADA in boys and girls (22.2% vs. 28%, p=0.76). Similar findings were observed by Glowinska et al. [49]. When patients in whom GADA was positive were compared with those in whom GADA was negative, there were no differences in their clinical features (frequency of DKA or BMI) or metabolic profile (HbA1c, insulin requirement or fasting C-peptide). Balasubramanian et al. in their cohort of 55 children (32 male and 23 female) with recent-onset T1DM also found no difference in clinical and biochemical characteristics between GADA-positive and -negative patients [6].

Idiopathic T1DM is a heterogeneous entity with numerous variants being described in the literature. For example, a fulminant form of T1DM has been described from Japan, China and Korea. The involvement of viral infection has been suggested as the pathogenesis of fulminant TIDM. It has a sudden onset with ketosis or ketoacidosis within a week after the onset of hyperglycemic symptoms Flu-like symptoms are observed in majority of the patients [3]. Winter et al. described idiopathic T1DM in young black Americans with obesity and acanthosis nigricans presenting with severe hyperglycemia and DKA [50]. However, presentations in our cohort did not resemble either of these variants and were similar to that described in non-Hispanic White Caucasian patients.

Our study also had certain limitations. The main limitations were a relatively small sample size. The study was done during the COVID-19 pandemic, where transport facilities and movement of the ordinary citizen was restricted due to lockdown restrictions. The tertiary healthcare systems were overloaded with COVID-19 cases which meant that not all the cases that would have otherwise reached came to the hospital. Secondly, we measured only GADA. However, in clinical practice, even in specialized centers, it is not suitable to screen all individuals of T1DM for all four islet-cell antibodies. Thirdly, we monitored our subjects for a short period after diagnosis of T1DM (0 to three months). Ongoing follow-up of this cohort will be important to determine the natural history of organ-specific autoimmunity in patients with T1DM.

## Conclusions

In summary, our study provide evidence that newly diagnosed T1DM patients should be screened routinely for organ-specific autoantibodies in particular TPOAb, tTGAb and PCA. Detection of these autoantibodies at onset may prevent complications associated with delayed diagnosis of these disorders. Three-fourths of our patients with T1DM were negative for GADA. While the absence of GADA did not impact the clinical and biochemical features in patients with T1DM, it may have relevance in the diagnosis of T1DM. Further studies are needed to unravel the clinical characteristics and etiology of GADA-negative (idiopathic) T1DM.
